# The “Gredouno” Cross Target: a new tool adapted to control *Glossina palpalis gambiensis* in the mangrove forests of Guinea

**DOI:** 10.1186/s13071-025-06783-2

**Published:** 2025-05-22

**Authors:** Kagbadouno Moïse, Camara Abdoulaye Dansy, Bart Jean-Mathieu, Solano Philippe, Bucheton Bruno, Camara Mamadou, Grébaut Pascal

**Affiliations:** 1 Programme National de Lutte contre les MTN à Prise en Charge des Cas, Ministère de la Santé et de l’Hygiène Publique, Conakry, Guinée; 2 INTERTRYP, Université de Montpellier, CIRAD, IRD, Montpellier, France

**Keywords:** *Glossina palpalis gambiensis*, Cross Target, Tiny Target, Efficacity, Mangrove

## Abstract

**Background:**

In the mangroves of Guinea, where the most active foci of human African trypanosomiasis in West Africa are located, vector control against tsetse flies using insecticide-impregnated Tiny Targets was first introduced in 2012. While annual deployments of Tiny Targets have resulted in an overall 90% reduction in tsetse fly densities in control areas, managing tsetse densities in specific biotopes such as mangrove channels, which are susceptible to significant climatic disturbances, presents greater challenges. Thus, a new three-dimensional model called the Cross Target was designed to address this situation.

**Methods:**

In the first phase, we evaluated the attractiveness of the Cross Target along with three other devices (the Tiny Target, the biconical trap, and the pyramidal trap) in a Latin square design. In a second phase, we assessed the efficacy of the Tiny Target and the Cross Target to control tsetse densities in a pilot field study led in two adjacent mangrove channels.

**Results:**

In the Latin square study, the Cross Target was significantly more attractive than the other devices, with a catch index of 2.23 (*P* = 0.03), 1.63 (*P* = 0.004), and 2.39 (*P* = 0.003) as compared with the biconical trap, the Tiny Target, and the pyramidal trap, respectively. In the pilot experimental field evaluation the Cross Target also showed its superiority, with tsetse density reduction reaching 90% 15 months after the initial deployment, whereas densities remained high in the channel where Tiny Targets were deployed.

**Conclusions:**

This study underscores the superiority of the new three-dimensional Cross Target in terms of attractiveness to tsetse flies and its resilience to climatic disturbances as compared with the conventional Tiny Target. The Cross Target presents a promising solution to enhance vector control effectiveness in challenging environments, such as mangroves or other hard-to-access areas, where target maintenance is particularly difficult.

**Graphical Abstract:**

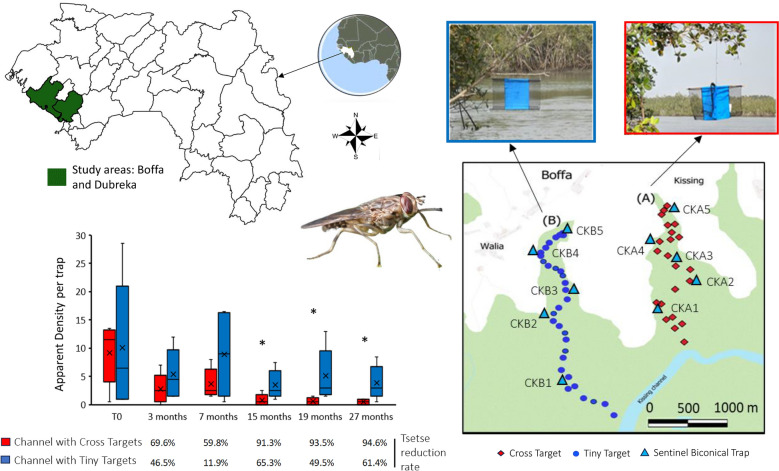

**Supplementary Information:**

The online version contains supplementary material available at 10.1186/s13071-025-06783-2.

## Background

Tsetse flies (*Diptera: Glossinidae*) are hematophagous insects belonging to the *Glossina* genus, and are cyclical vectors of trypanosomes, which cause both human and animal trypanosomoses in Africa. These diseases still represent a major constraint on public health, rural development, agriculture, and livestock breeding in sub-Saharan Africa. Among the 31 known species and subspecies, only 2 (*G. palpalis* and *G. fuscipes*) are currently involved in transmitting gambiense human African trypanosomiasis (g-HAT).

Tsetse control has always been pivotal in the fight against animal trypanosomoses. For g-HAT tsetse control, insecticide-impregnated traps or targets [1] have been used with success in Cote d’Ivoire, Uganda, and Congo, but were then abandoned, with most of the control efforts being directed toward disease diagnostics and treatments of patients since the 1990s. This began to change more than a decade ago with the design of a new, small-sized two-dimensional deltamethrin-impregnated pieces of cloth called “Tiny Targets,” which greatly facilitated the logistics and costs of deployment by local teams [2, 3]. In all the active g-HAT foci where Tiny Targets have been deployed, in combination with diagnostics and treatments of cases, they have accelerated the interruption of transmission, including in Chad [4, 5], in Cote d’Ivoire [6], in Guinea, in Uganda, and in DRC, as reviewed in [7, 8].

In Guinea, this device was first introduced in 2012 in the mangrove focus of Boffa, in addition to mass screening activities, and was shown to significantly reduce parasite transmission and to speed up the disease elimination process [9]. Since 2018, up to 15,000 targets have to be deployed each year in coastal Guinea to cover the three active disease foci. With less than one case detected per 10,000 inhabitants in endemic districts, the elimination threshold as a public health problem was reached in 2023. Nevertheless, HAT cases (around 20 per year) are still being diagnosed passively or actively by the national control program [10], indicating that residual transmission to humans is still persisting.

Controlling tsetse flies in this mangrove biotope is particularly challenging for many reasons. Population genetics studies of *G. palpalis gambiensis*, the main g-HAT vector in coastal Guinea, have revealed large panmictic population sizes with high dispersal rates in comparison with those observed with other *Glossina* species living in riverine habitats [11]. Large areas of the mangrove are also inaccessible, human populations are very mobile, and Tiny Target deployments are focused on sites frequented by the human population to lower human–tsetse contacts and halt transmission, but are prone to constant re-invasion from the inside. This is particularly true in medium-sized mangrove channels where tsetse densities are high and where Tiny Targets have to be hung on mangrove tree branches where they are highly exposed to climatic disturbances such as the wind and/or high tide coefficients [12]. As a consequence, almost half of the Tiny Targets become nonfunctional a few months after their deployment, impairing their full efficacy in this specific biotope (M.K. personal communication). To address this issue, a new three-dimensional target model composed of two Tiny Targets sewn together and a unique hanging point (the “Gredouno” or Cross Target) has been designed.

The aim of our study was to evaluate this new model in comparison with the Tiny Target and other trapping devices used to evaluate tsetse fly densities and to contribute to the improvement of tsetse control tools to accelerate the process of stopping g-HAT transmission in mangrove areas by 2030.

## Methods

### Study area

**Fig. 1:**
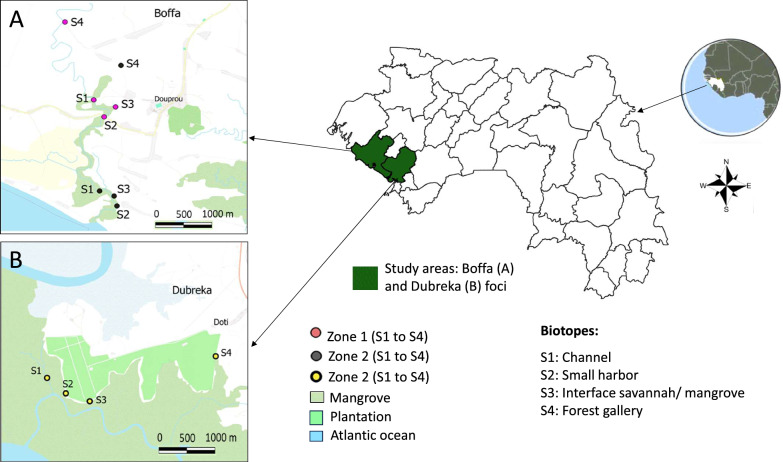
Location of study areas for the Latin square experiments

This study took place in coastal Guinea, in the mangrove zone that hosts the currently active foci [13, 14]. Our experiments were carried out in the prefectures of Boffa and Dubréka (10°32’21’’ N and 13°22’30’’ W) (Fig. 1). Maritime Guinea represents a 300 km strip stretching from Guinea Bissau in the north to Sierra Leone in the south [15]. The landscape has two distinct parts: a continental part, characterized by sandstone soil and a plant formation dominated by *Elaeis guineensis*; and an island part, made up of mangroves (a marine aquatic formation) that is subject to varying ocean tides. The vegetation is dominated by two halophilic species: *Rhizophora* spp. and *Avicennia* spp. The mangrove is irrigated by saline sea water through several channels called mangrove channels. The wild fauna is composed of a large diversity of mammals, birds, and reptiles species [16]. Livestock does not constitute an important part of the economy and consists mainly of small ruminants (goats and sheep), whereas cattle and pig breeding are restricted to a few households [14]. The climate is humid tropical, with two 6-month seasons: a dry season from November to April and a rainy season from May to October. Average annual rainfall can reach up to 3450 mm, and average temperatures vary between 24 °C and 30 °C, with a maximum in April and a minimum in August. Maximum relative humidity is 97% in August [17].

### Tsetse control devices used in the study

**Fig. 2:**
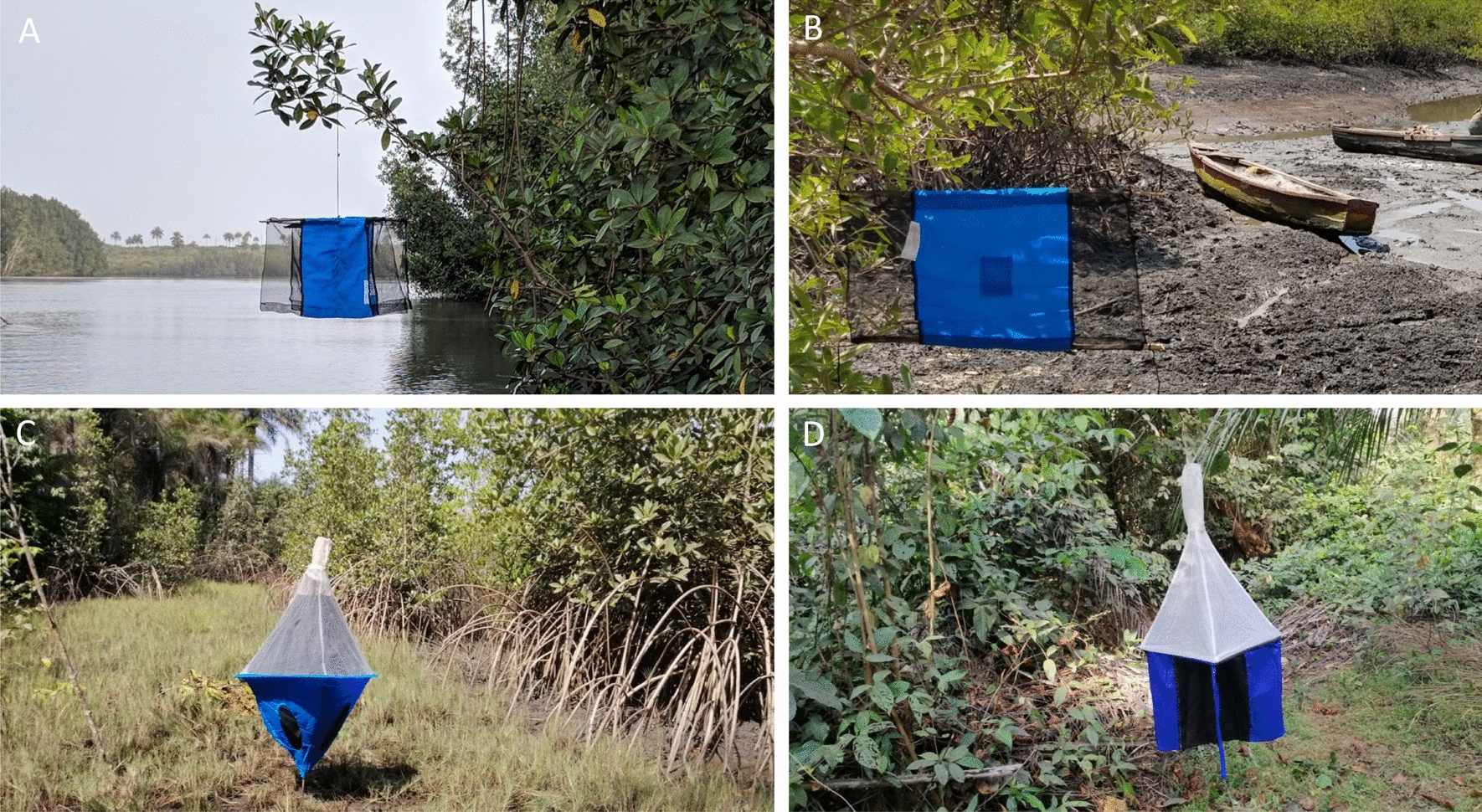
The four models of devices used during our study in four different biotopes. Cross Targets in a mangrove channel (**A**), Tiny Targets in a harbor (**B**), Biconical traps in a mangrove–savanna interface (“fassadé”) (**C**), and pyramidal traps in a forest gallery (**D**)

Four entomological devices used for the control of tsetse flies, characterized by differently shaped pieces of blue and black cloth, were used in the framework of this study (Fig. 2): (A) the Cross Target; (B) the Tiny Target; (C) the biconical trap; and (D) the pyramidal trap.

The Tiny Target and the biconical traps were manufactured and supplied by Vestergaard Frandsen Ltd. The Cross Target is made from two Tiny Targets sewn together. This material consists of 100% polyester and 100% polyethylene fabrics. The pyramidal trap is made of cotton.

The Tiny Target (Fig. 2B; Supplementary Fig. 1) is black–blue–black, 50 cm high and 75 cm wide [18], and consists of a central strip of pthalogen blue fabric and two side strips of black mosquito netting. A nylon thread is attached to each of the two upper-ends of the target to attach the device to two wooden stakes or branch supports.

The Cross Target (Fig. 2A; Supplementary Fig. 1) results from the junction of two Tiny Targets sewn at a 90° angle, giving the cross shape of the target. The connecting seam is made in the central part (pthalogen blue fabric) of each Tiny Target and is located 37.5 cm from the sides. The cross shape thus gives four identical portions, giving it a three-dimensional function. Every portion has a vertical side-strip of black mosquito netting and a central strip of blue pthalogen fabric, as with the Tiny Target (black–blue–black).

**Fig. 3:**
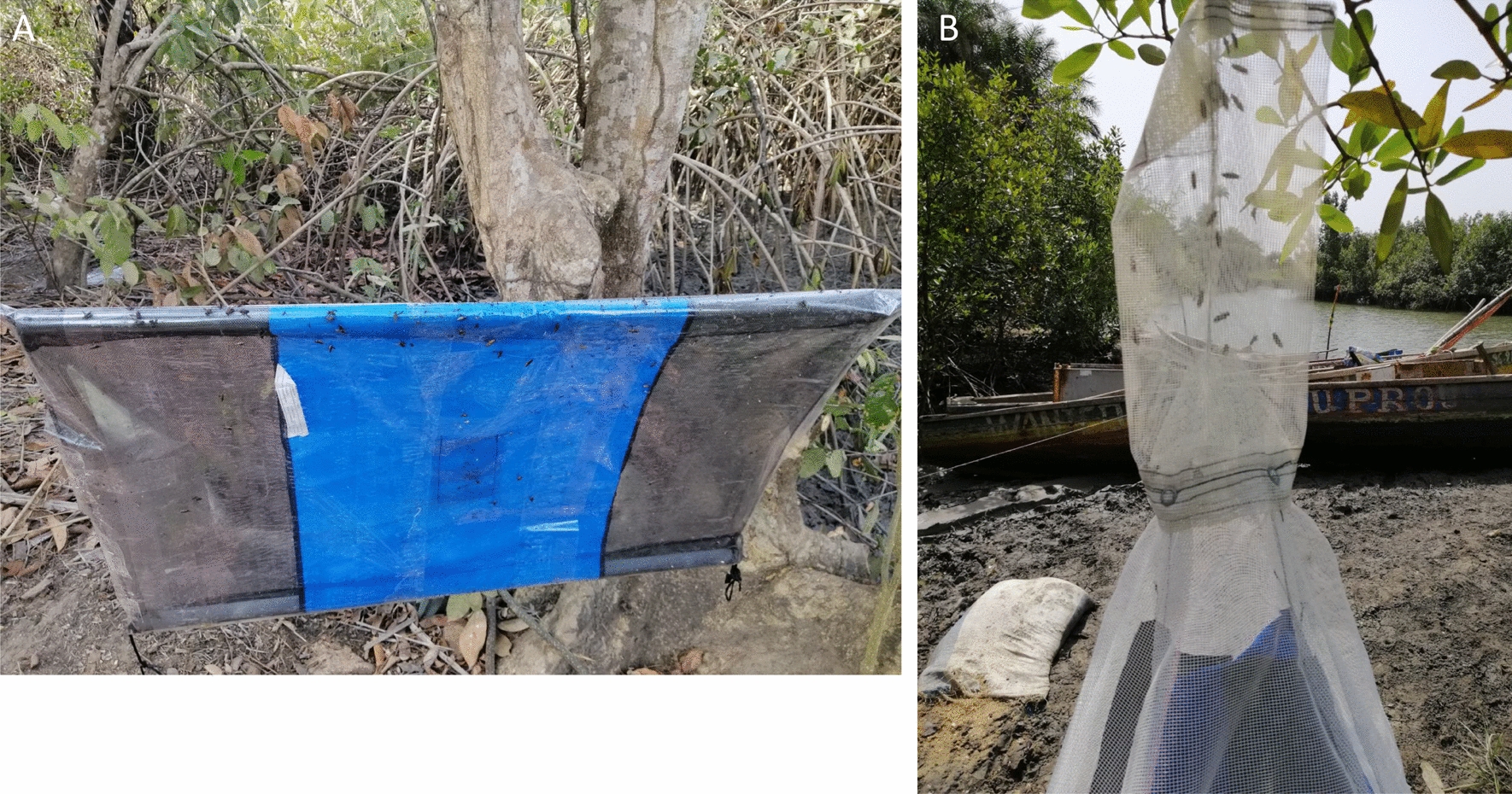
Tsetse fly capture devices. Tiny Target covered with adhesive on which tsetse flies are held (**A**). Cage on top of a trap in which tsetse flies are caught (**B**)

To enable tsetse flies to be captured with these two models of targets for the Latin square experiments, a double adhesive film (Rentokil Initial Supplies, UK) was used to cover all the surfaces (Fig. 3A) to retain any flies landing on them [19, 20].

The biconical trap (Fig. 2C) [21] comprises an attractive part, made of blue pthalogen fabric sewn into a conical shape, and through three openings created in this blue fabric a second part made of black polyamide fabric. A third part, made of white mosquito netting with a small hole at the top, is covered by a cage to trap tsetse flies (Fig. 3B). The conical shape is maintained by a tightly stretched metal hoop and the trap is mounted on a metal bracket.

The pyramid trap (Fig. 2D) [22] has a monoconic shape, simplified for easy and economical use, and is made up of four side strips in pthalogen blue and a central strip in black polyamide fabric. The top of these strips is fitted with white mosquito netting in the shape of a pyramid. This shape is created by two wooden slats mounted on each edge. A cage is attached to the top of the clear netting to trap the tsetse flies. The trap is suspended from a tree branch by a rope attached to the cage (Fig. 2D and 3B).

### Latin square evaluation of the attractiveness of tsetse control devices

For the Latin square experiments, three areas, all located outside of the vector control area, were identified: two in the Doprou area in Boffa (area 1 and area 2) and one in the Doti area in Dubreka (area 3). For each zone, four trapping sites were chosen: one in a mangrove channel, one in a mangrove harbor, one at the mangrove–savanna interface, and one in a forest gallery, each of which are representative of the different biotopes encountered in mangrove areas (Fig. 2). The experiment was based on an n × n grid of columns and rows in a square table. The scheme obeys the random arrangement of the elements in this table so that each element can occupy any of the columns and rows in a given replication (Supplementary Fig. 2). The first Latin square experiment was carried out in the Doprou area in April 2021 with the four devices. The first replicate was made in area 1 (days 1–4), a second replicate in area 2 (days 5–8), and the third replicate again in area 1 (days 9–12), so that for each device we had triplicate measurements for each biotope (mangrove channel, mangrove harbor, mangrove–savanna interface, and forest gallery: 12 measurements per device in total). The second Latin square experiment was carried out in the Doti area (Dubreka) in November 2022 (zone 3) and concerned only the Cross Target and the Tiny Target. In this experiment, the three replicates were performed at the same sites on 12 consecutive days (Supplementary Fig. 2).

### Pilot comparative evaluation of the efficacy of the Cross Targets and Tiny Targets in the field

In the vector control zone of Kissing (Boffa), where tsetse densities remained high after 3 years of control, two ecologically similar mangrove channels, each around 3000 m long, were identified (Fig. 5). A total of 22 Cross Targets were deployed to cover channel A and 32 Tiny Targets were deployed in channel B and were replaced after 1 year. The entomological evaluations were carried out at five sentinel sites located along each channel with biconical traps used for 48 h and collected each day. The first entomological evaluation took place just before the first deployment (T0) and was repeated at 3, 9, 15, 19, and 27 months post initial deployment. The state of the targets (normal, submerged, folded, or absent) was also recorded upon each evaluation.

### Statistical analysis

The JMP software (JMP^®^ 11, SAS Institute Inc. 2013) was used for all statistical analyses and Microsoft Excel (2016) was used to generate graphs.

#### Latin square study

The first analysis of tsetse daily catches in the Latin square datasets showed that the distribution did not follow a normal distribution. All subsequent statistical analyses were thus carried out on log (X + 1) transformed data. To compare the different devices tested in the Latin square study we used linear regression models in which daily tsetse catches were weighted by the day of capture and in which the trapping site, the biotope, and the different devices were included as explanatory covariates in the multivariate analysis. For better clarity, mean daily catches in linear models are presented as detransformed data.

#### Pilot assessment of Cross Targets and Tiny Targets in the field

In order to assess the efficacy of the Cross Targets versus Tiny Targets in the mangrove channels where they were deployed, the Student’s *T*-test was used to compare the mean daily catches at each entomological evaluation point, or for each device at each time point, in comparison with T0. Statistical analyses were carried out on log (X + 1) transformed data but are shown as daily catches on graphs.

## Results

### Experiment 1

**Fig. 4:**
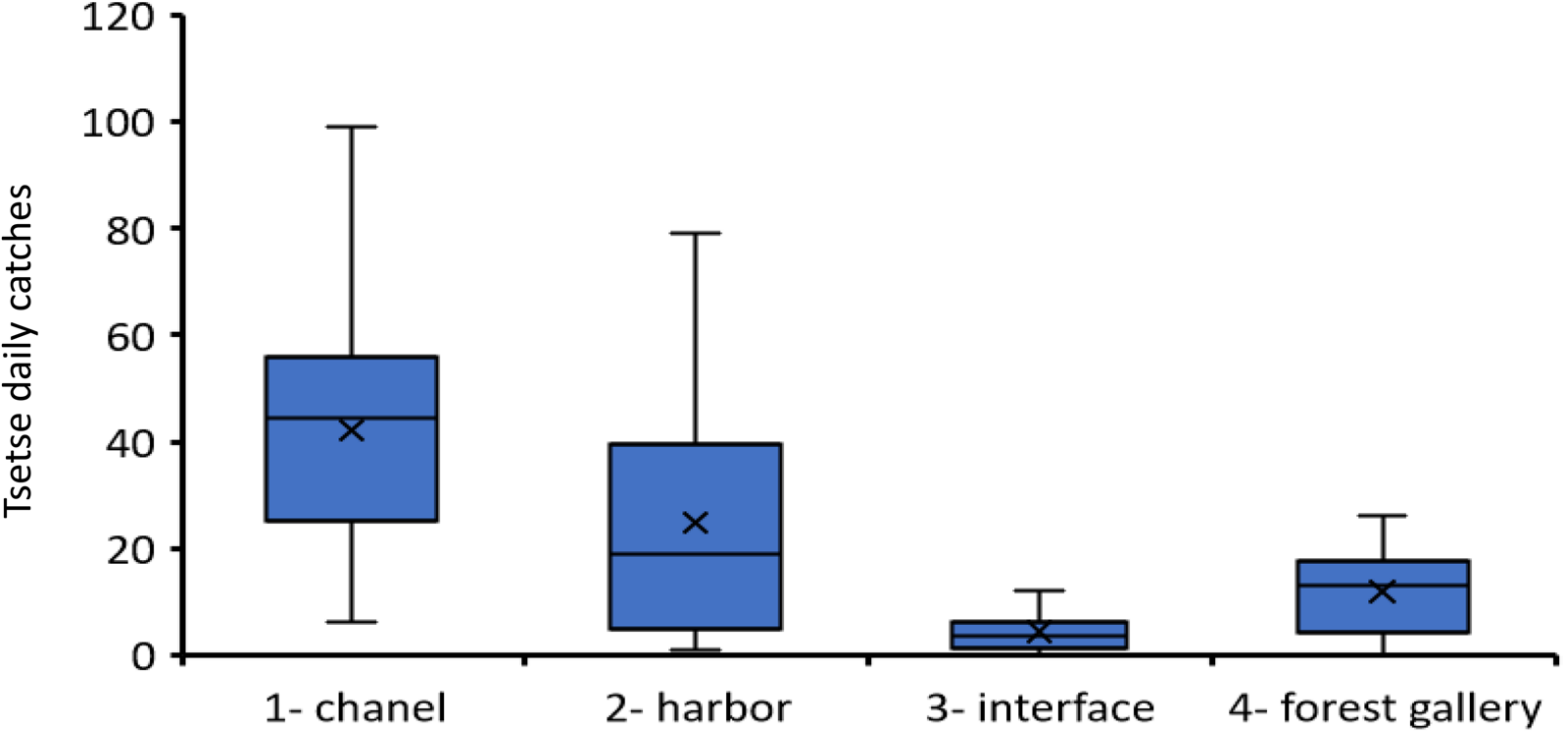
Box plot of tsetse daily catches in the different biotopes

The first Latin square experiment was designed to compare the attractiveness of the Cross Target and Tiny Target along with two other trapping devices (the biconical trap and the pyramidal trap) that can be used for the entomological evaluation of tsetse fly densities. The experiment was conducted in the Doprou area, where no tsetse control measures are being carried out and included four types of sites (mangrove channels, mangrove harbors, mangrove–savanna interfaces, and forest galleries along rivers) that are representative of the different biotopes encountered in mangrove areas. As shown in Fig. 4, the biotope was significantly associated with the daily tsetse catches, with the highest densities observed in mangrove channels (mean daily catches = 66.1), pirogue jetties (mean daily catches = 51.8), forest galleries (mean daily catches = 19.4), and mangrove–savanna interfaces (mean daily catches = 8.6).

In the subsequent analysis to compare the different devices, the number of flies trapped per day was adjusted on the day of trapping, and the biotope and the replicate site of capture were included as covariates in the multivariate linear regression analysis (Table 1).

**Table 1:** Multivariate logistic regression model of tsetse catches according to the different devices used in the study (first Latin square experiment in Boffa)

*R*^2^ model = 0.65	*P* < 0.0001			
Covariates	*P*-value^a^	Mean catches per day	Catch index	*P*-value^b^
Trapping site	0.016	–		
Biotope	< 0.0001	–		
Device	0.016	–		
Cross Target		23.9 [14.2–33.9]	1	
Tiny Target		10.7 [6.6–15.7]	**0.45**	**0.004**
Biconical trap		14.6 [8.9–21.3]	**0.61**	**0.03**
Pyramidal trap		10.0 [5.4–12.9]	**0.42**	**0.003**

*P* values < 0.05 are indicated in bold

^a^*P*-values calculated for the whole model including the four devices

^b^*P*-value calculated for 2 × 2 comparisons with the Cross Target as reference

The Cross Target was the best trapping device, with tsetse fly catch indexes of 0.61, 0.45, and 0.42 for biconical traps (*P* = 0.03), Tiny Targets (*P* = 0.004), and pyramidal traps (*P* = 0.003), respectively.

### Experiment 2

The second Latin square experiment was designed to extend the comparison of the Cross Target versus the Tiny Target in another site located in another mangrove focus (Dubreka) and to increase the statistical power to compare these two devices in the different ecological biotopes encountered in mangrove HAT foci (Table 2).

**Table 2:** Multivariate logistic regression model of tsetse catches by the Cross Targets and Tiny Targets according to different biotopes (first and second Latin square experiments)

*R*^2^ model = 0.77	*P* < 0.0001			
Covariates	*P*-value	Mean catches per day cross targets	Mean catches per day tiny targets	Catch index*
Trapping site	0.003			
Biotope	< 0.0001			
Device	< 0.0001	38 [26.5–48.1]	12.7 [9.4–17]	**3.0**
Mangrove channel	0.02	89.3 [55.3–133.1]	42.9 [25.1–64.8]	**2.1**
Mangrove harbor	0.02	77 [43.3–123.4]	26.6 [14.3–40.8]	**2.9**
Mangrove–savanna interface	0.34	9.7 [5.6–15.4]	7.5 [4.2–11.5]	1.3
Forest gallery	0.001	34.6 [15.8–71]	4.2 [1.9–7.6]	**8.2**

*P* values < 0.05 are indicated in bold

^*^The catch index is calculated with the Tiny Target set as the reference

 The superiority of the Cross Target over the Tiny Target was confirmed in this experiment (catch index = 3; *P* < 0.0001) and further indicated that the observed differences were the highest for forest galleries (catch index = 8.2), followed by that in mangrove harbors (catch index = 2.9), and then mangrove channels (catch index = 2.1), whereas no significant differences were observed at the mangrove–savanna interface.

### Pilot assessment of Cross Targets and Tiny Targets in the field

**Fig. 5:**
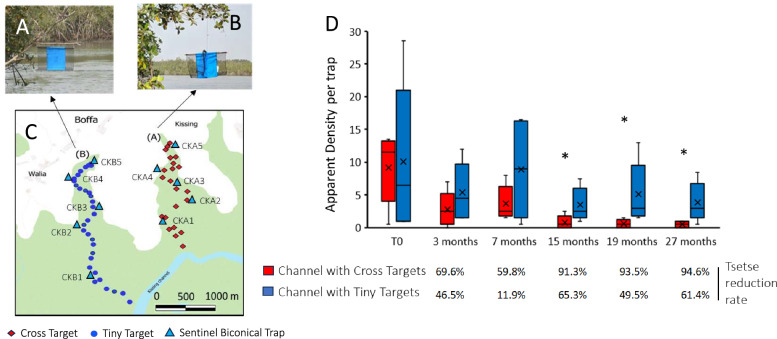
Evolution of tsetse densities according to the type of targets. Tiny Targets (**A**) and Cross Targets (**B**) were deployed along two channels in Boffa (**C**). Periodically, for 27 months, tsetse flies were captured with sentinel biconical traps to assess tsetse fly apparent density (**D**). * indicates a significant difference between apparent density in the channel with Tiny Targets compared with the channel with Cross Targets. Significant reduction rates in comparison with T0 are bolded

**Fig. 6:**
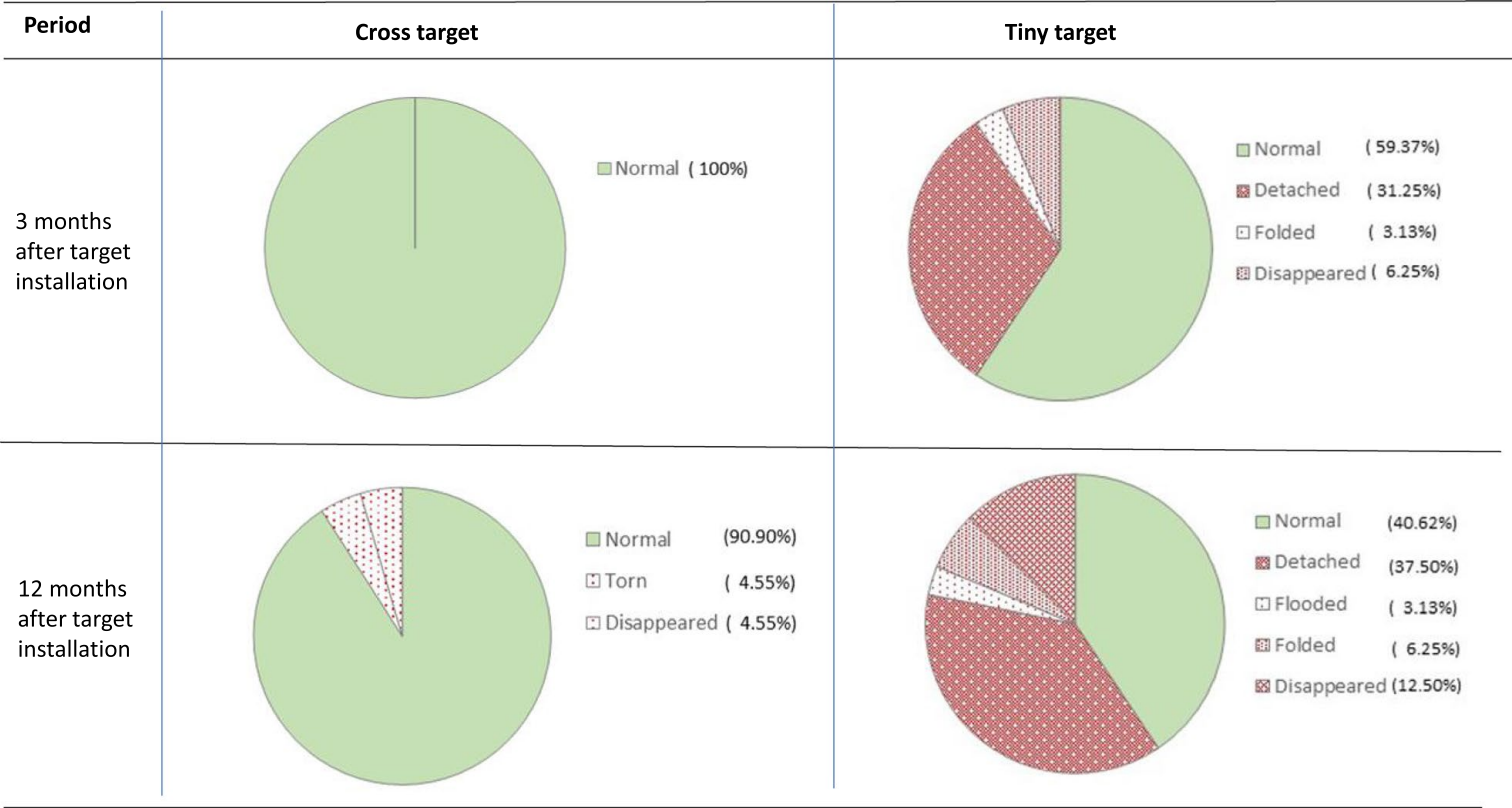
Resistance of the material according to the model under field conditions

Finally, two adjacent mangrove channels, where a limited impact of the vector control measures were observed during the entomological evaluation of the National Control Program, were selected for a pilot study involving the annual deployment of either Tiny Targets or Cross Targets (Fig. 5). In the mangrove channel where the Cross Targets were deployed, a significant reduction of more than 90% was observed after 12 months and was maintained until the end of the study. In contrast, the reduction of tsetse densities was only at a maximum of 50% and was not significantly different from the densities measured at T0 in the channel where Tiny Targets were deployed. A major difference between the two study sites was the number of devices that remained functional over time. Three months after the initial deployment, all the Cross Targets (*n* = 22) were found in situ and in good condition, while only 59% (*n* = 19/32) of the Tiny Targets were found in normal condition; with the remaining 41% (*n* = 13/32) as missing (6%, *n* = 2), detached (31%, *n* = 10), or folded (3%, *n* = 1) (Fig. 6). After 1 year, the number of anomalies remained minimal for the Cross Targets (*n* = 2/22), whereas only 40% (*n* = 12/32) of Tiny Targets remained fully functional. The main anomalies recorded for Tiny Targets were the detachment from one of the two hanging sites (37.5%) and the total disappearance of the target (12.5%).

## Discussion

Although vector control through the deployment of Tiny Targets has recently shown its efficacy in accelerating the elimination of HAT, controlling tsetse flies in complex biotopes, such as mangroves, can be challenging. We herein report a new tsetse control device, the Cross Target, made by combining two Tiny Targets, which was shown to be more attractive and resilient to climatic disturbances.

In the combined Latin square analysis (Boffa and Dubreka), the Cross Target appeared to be three times more attractive than the Tiny Target. This was especially true in biotopes where the space was enclosed by vegetation (mangrove trees along channels or riverine forest along water streams), but the difference was not significant at the more open spaces seen at mangrove–savanna interfaces. This new target is simply composed of two Tiny Targets sewn together at a 90° angle. The fact that the surface of tissue is doubled as compared with the Tiny Target may of course partly account for its better attractiveness, but is probably not the only factor explaining this. In fact, the 2D visible surface of the Cross Target is the same as the Tiny Target (50 cm × 75cm), with the same order of black–blue–black strips. The main difference is that as a tri-dimensional object, all of the surfaces remain visible for flies located on the sides, thus increasing the surface (and maybe the distance) from which it is visible (Supplementary Fig. 3). Furthermore, having a unique hanging point, the Cross Target turns on itself with the wind and this movement may also greatly increase its attractiveness. In contrast, the Tiny Target is fixed on its two upper sides, preventing it from turning over and its bi-dimensional form reduces its visibility from the sides (Supplementary Fig. 4).

Importantly, the Cross Target was also shown to be able to induce a significant reduction of tsetse fly densities (up to 90%) in medium-sized mangrove channels, where the effect of the Tiny Target seems to be limited. In areas where tsetse densities are particularly high and subject to constant re-invasion from untreated areas, Tiny Targets are effective in suppressing the densities of tsetse flies to around 5–10 flies/trap/day but are not enable to reach higher suppression levels. In such a context, our results show that the usage of the Cross Target can be useful to achieve better control of tsetse populations. An important factor here, in addition to the high attractiveness, is that the Cross Target was shown to be more resilient to climatic disturbances. Most Cross Targets were found to remain functional 1 year after their initial deployment, whereas over half (59%) of the Tiny Targets had been disturbed by the same time point. While the maintenance of Tiny Targets can easily be done by the population on the continental part of the focus, maintaining/replacing Tiny Targets in mangrove channels requires important logistic planning. Because of its higher cost, the Cross Target is unlikely to replace Tiny Targets in controlling tsetse flies in Guinea, but could be used in a targeted manner in mangrove channels that are difficult to access and in mangrove harbors that are especially risky sites for transmission/diffusion of the disease due to population movements and gatherings at these specific places.

Interestingly, the Cross Target covered with an adhesive film also appeared to be more effective in capturing tsetse flies than the biconical trap, known to be one of the best devices to capture tsetse flies from the *Glossina palpalis* and *gambiensis* sub-species [21, 23]. In Guinea, the biconical trap is routinely used for entomological evaluation surveys designed to follow the evolution of tsetse fly densities. In contexts where the tsetse fly densities become very low, the Cross Target with adhesive films could thus be used as an alternative/complementary, more sensitive tool, to document the absence of tsetse flies in such areas.

## Conclusions

In our experimental study, we have shown the superiority of the Cross Target over the Tiny Target both in terms of attractiveness and resistance to climatic disturbance in mangrove biotopes. This new device can thus be used as an alternative to Tiny Target deployments to improve tsetse control in mangroves (mangrove channels and mangrove harbors in the specific context of Guinea) or in other remote biotopes that are difficult to access and where the maintenance of targets over time is difficult.

## Supplementary Information

Additional file 1: Figure 1. Assembly drawing of the Cross Target from two Tiny Targets. Figure 2. Description of the Latin square design. Figure 3. Schematic representation of the visibility of the Cross Target to tsetse flies in a mangrove channel. Figure 4. Schematic representation of the visibility of the Tiny Target to tsetse flies in a mangrove channel.

## Data Availability

No datasets were generated or analyzed during the current study.
