# Supplementary figures and images for: The “Gredouno” Cross Target: a new tool adapted to control Glossina palpalis gambiensis in the mangrove forests of Guinea
Source: Parasit Vectors. 2025 May 22;18:185. doi: 10.1186/s13071-025-06783-2 (PMC12096635; doi:10.1186/s13071-025-06783-2)

**Supplementary Materials**

**Suppl Figure 1**


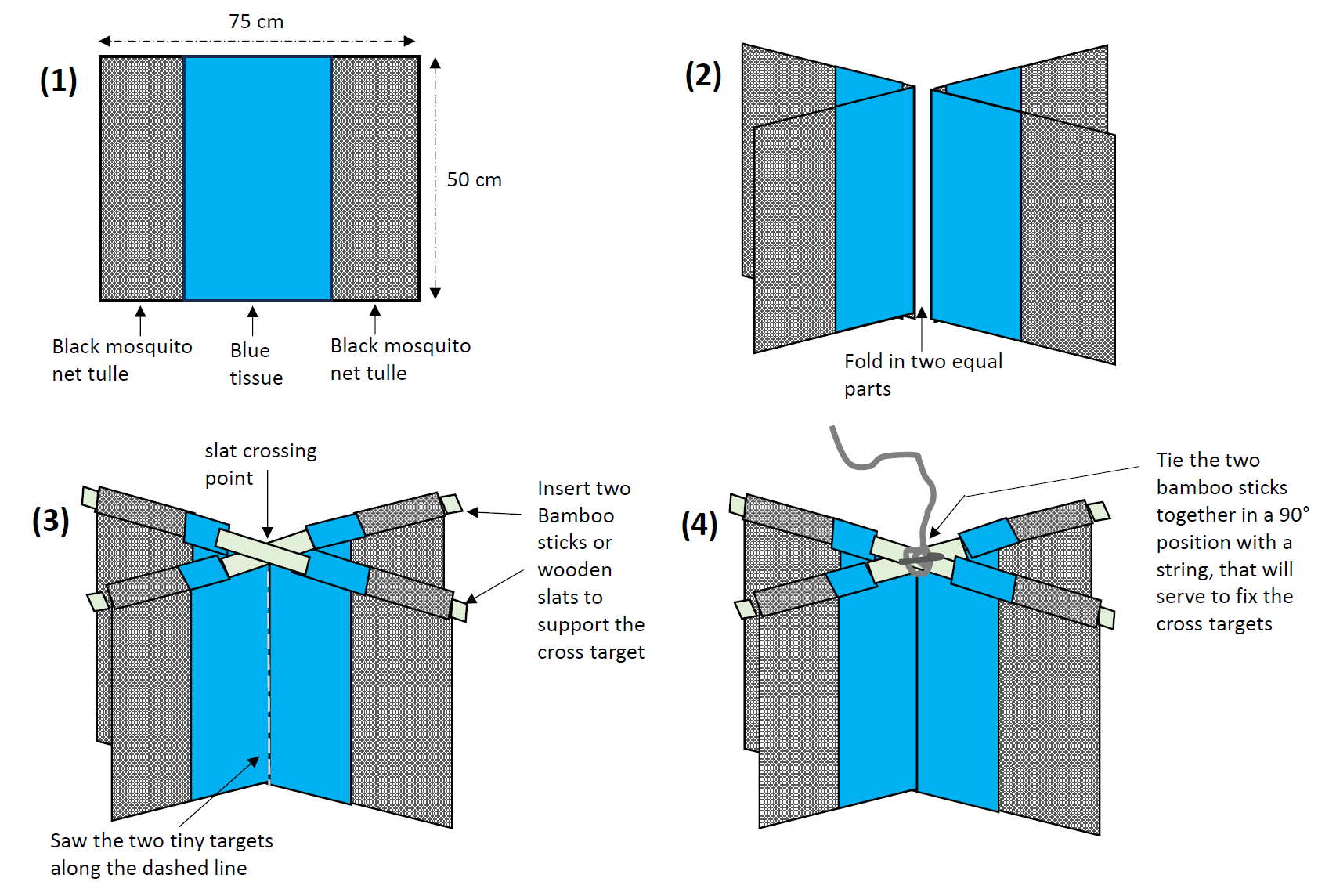


**Suppl Figure 2**


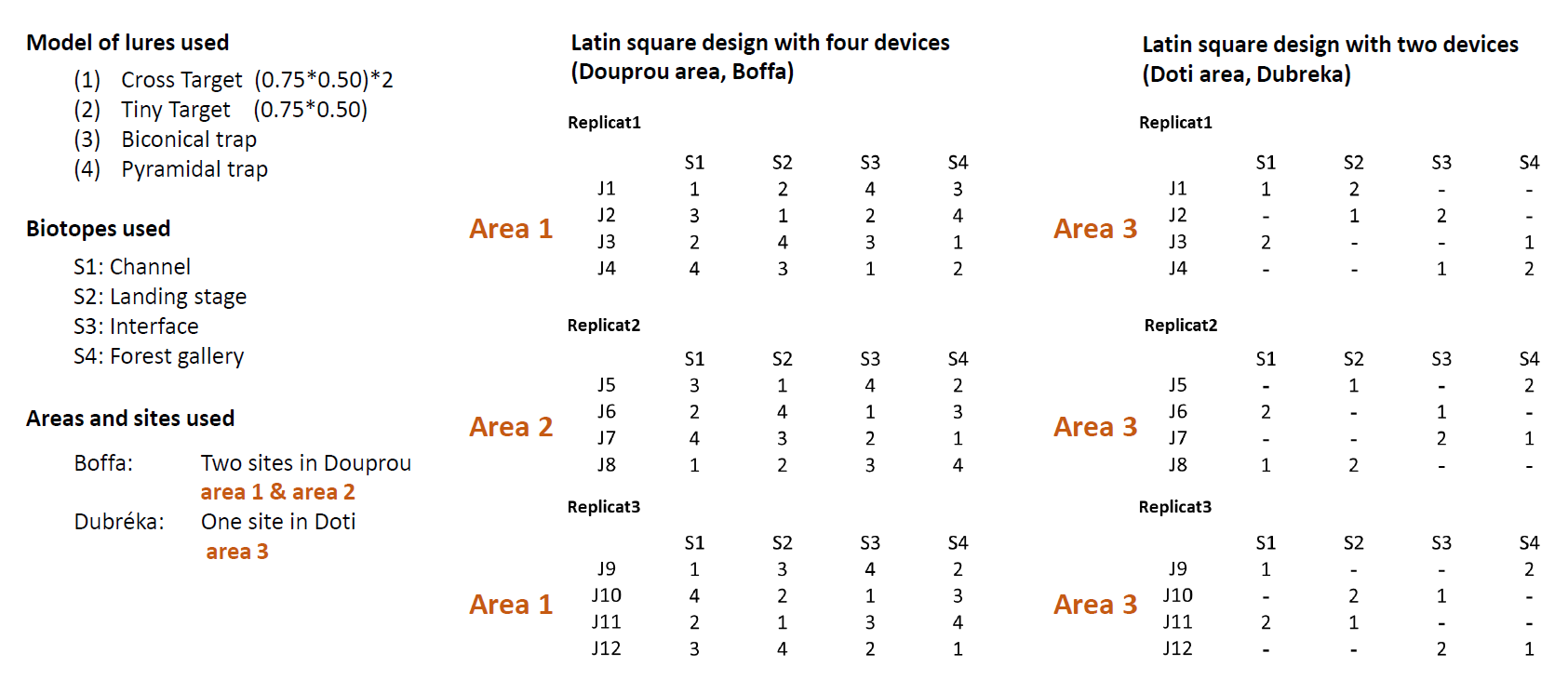


**Suppl Figure 3**


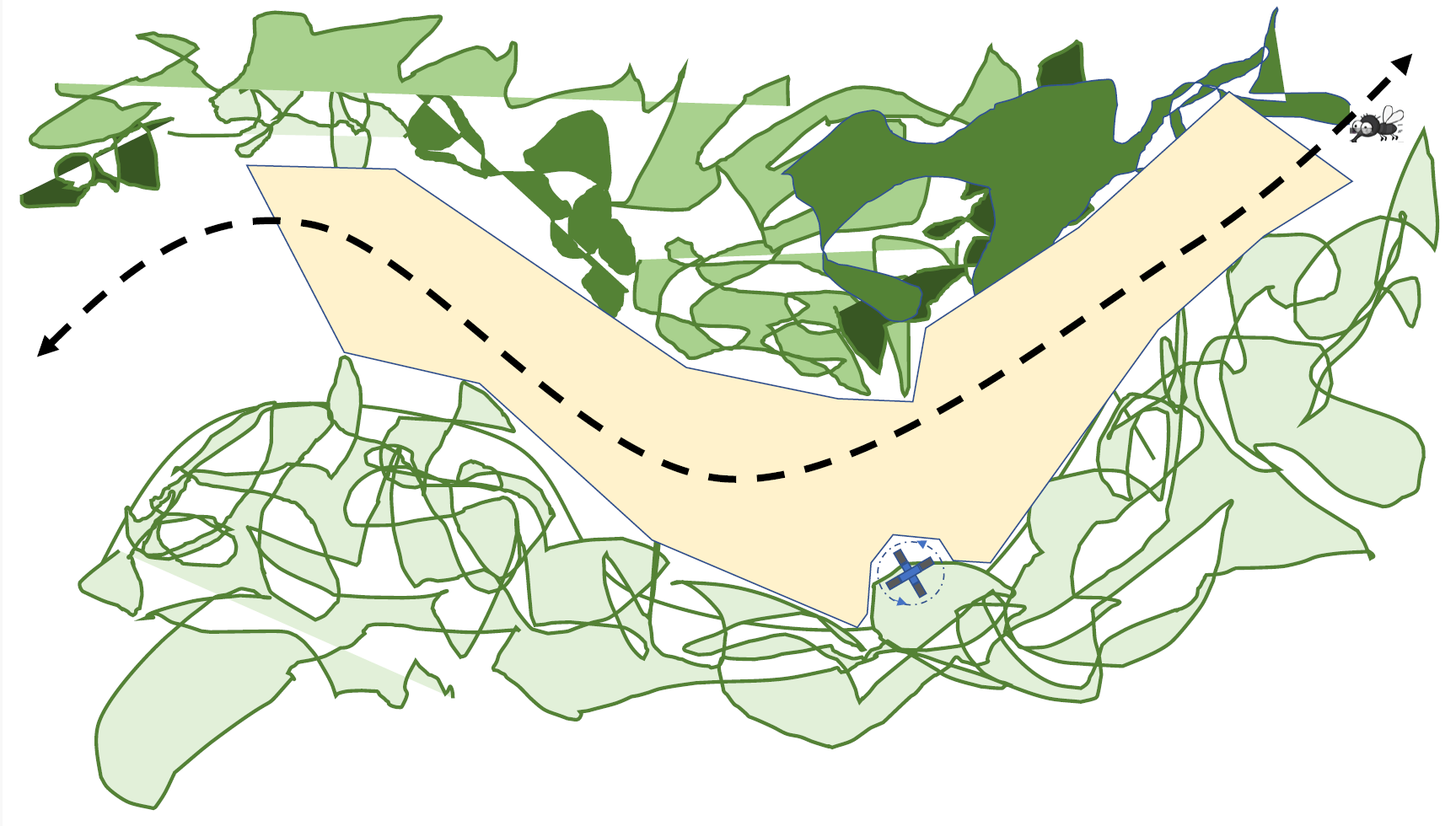


**Suppl Figure 4**


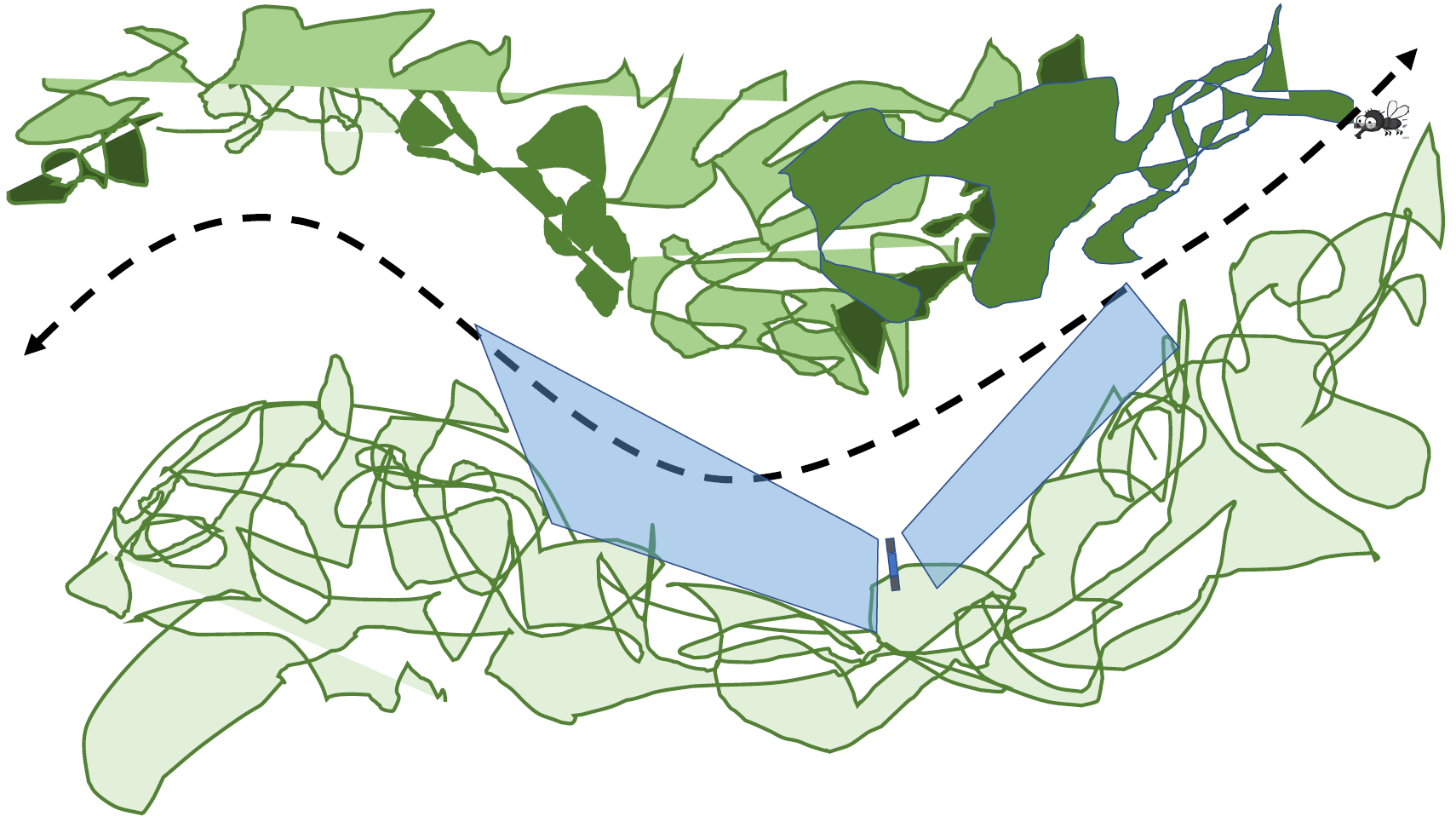

Supplement: Supplementary file 1 — Additional file 1: Figure 1. Assembly drawing of the Cross Target from two Tiny Targets. Figure 2. Description of the Latin square design. Figure 3. Schematic representation of the visibility of the Cross Target to tsetse flies in a mangrove channel. Figure 4. Schematic representation of the visibility of the Tiny Target to tsetse flies in a mangrove channel. [file 13071_2025_6783_MOESM1_ESM.docx]
